# Feasibility of feeding *Aedes aegypti* mosquitoes on dengue virus-infected human volunteers for vector competence studies in Iquitos, Peru

**DOI:** 10.1371/journal.pntd.0007116

**Published:** 2019-02-12

**Authors:** Kanya C. Long, Juan Sulca, Isabel Bazan, Helvio Astete, Hugo L. Jaba, Crystyan Siles, Claudine Kocher, Stalin Vilcarromero, Julia Schwarz, Karin S. Escobedo-Vargas, Fanny Castro-Llanos, Leslye Angulo, Guadalupe Flores, Cesar Ramal-Asayag, Eric S. Halsey, Robert D. Hontz, Valerie A. Paz-Soldan, Thomas W. Scott, Louis Lambrechts, Amy C. Morrison

**Affiliations:** 1 Department of Entomology and Nematology, University of California, Davis, Davis, California, United States of America; 2 Virology and Emerging Infections Department, U.S. Naval Medical Research Unit No. 6, Washington DC, Lima and Iquitos, Peru; 3 Entomology Department, U.S. Naval Medical Research Unit No. 6, Washington DC, Lima and Iquitos, Peru; 4 Icahn School of Medicine at Mount Sinai, New York, New York, United States of America; 5 Department of Internal Medicine, Loreto Regional Hospital “Felipe Santiago Arriola Iglesias,” Punchana, Iquitos, Peru; 6 School of Medicine, Universidad Nacional de la Amazonia Peruana, Iquitos, Peru; 7 Global Community Health and Behavioral Sciences Department, Tulane University School of Public Health and Tropical Medicine, New Orleans, Louisiana, United States of America; 8 Insect-Virus Interactions Group, Department of Genomes and Genetics, Institut Pasteur, Paris, France; 9 Centre National de la Recherche Scientifique, Unité Mixte de Recherche 2000, Paris, France; University of Heidelberg, GERMANY

## Abstract

**Background:**

Transmission of dengue virus (DENV) from humans to mosquitoes represents a critical component of dengue epidemiology. Examinations of this process have generally been hampered by a lack of methods that adequately represent natural acquisition of DENV by mosquitoes from humans. In this study, we assessed artificial and natural blood feeding methods based on rates of DENV infection and dissemination within mosquitoes for use in a field-based epidemiological cohort study in Iquitos, Peru.

**Methodology/Principal findings:**

Our study was implemented, stepwise, between 2011 and 2015. Participants who were 5 years and older with 5 or fewer days of fever were enrolled from ongoing clinic- and neighborhood-based studies on dengue in Iquitos. Wild type, laboratory-reared *Aedes aegypti* were fed directly on febrile individuals or on blood collected from participants that was either untreated or treated with EDTA. Mosquitoes were tested after approximately 14 days of extrinsic incubation for DENV infection and dissemination. A total of 58 participants, with viremias ranging from 1.3 × 10^2^ to 2.9 × 10^6^ focus-forming units per mL of serum, participated in one or more feeding methods. DENV infection and dissemination rates were not significantly different following direct and indirect-EDTA feeding; however, they were significantly lower for mosquitoes that fed indirectly on blood with no additive. Relative to direct feeding, infection rates showed greater variation following indirect-EDTA than indirect-no additive feeding. Dissemination rates were similar across all feeding methods. No differences were detected in DENV infection or dissemination rates in mosquitoes fed directly on participants with different dengue illness severity.

**Conclusions/Significance:**

Our study demonstrates the feasibility of using direct and indirect feeding methods for field-based studies on vector competence. Direct mosquito feeding is preferable in terms of logistical ease, biosecurity, and reliability.

## Author summary

In the context of ongoing clinic- and field-based epidemiological studies on dengue virus (DENV), we compared methods of feeding mosquitoes on blood from naturally infected humans. Participants chose to participate in direct (uninfected mosquitoes applied directly to skin) and/or indirect (uninfected mosquitoes fed on a membrane feeder in the laboratory with blood drawn from a participant) methods. Overall, rates of DENV infection and dissemination were lower in mosquitoes fed indirectly on blood with no additive than in mosquitoes fed directly on a participant. Rates of DENV infection and dissemination were similar between mosquitoes fed directly and indirectly when anticoagulant (EDTA) was added. The indirect-EDTA method resulted in more variable infection rates than the direct method. Our results demonstrate the feasibility of using either direct or indirect feeding methods to study DENV-human infectiousness to mosquito vectors. In our experience, however, direct feeding is preferable to indirect feeding in terms of logistical ease, biosecurity, and reliability.

## Introduction

Mosquito-borne viruses are widespread and increasingly important to public health. Since 2013, two of these viruses, chikungunya and Zika viruses, have expanded into the Western hemisphere and been associated with a wider range of pathologies than previously recognized, greatly increasing their public health impact[1, 2]. These viruses have now been reported in Africa, Asia, and the Americas, with small-scale outbreaks of chikungunya in Europe. Dengue virus (DENV) remains the most prevalent of the mosquito-borne viruses, with patterns of disease varying geographically based on the co-circulation of serotypes 1–4, different virus genotypes, human population growth and migration, including urbanization [3], and geographic expansion of mosquito vectors [4]. Understanding the epidemiology of mosquito-borne viruses is complicated by the need to quantify virus transmission between humans and mosquitoes. While infections in humans have been extensively studied in clinic- and community-based studies, examining natural transmission of DENV from humans to mosquitoes and the potential for mosquitoes that feed on DENV-infected people to contribute to onward transmission has faced challenges related to methods and perceived acceptability [5].

Methodologically, examinations of the infectiousness of virus to mosquitoes have had important experimental constraints that limit extrapolation to natural human-to-mosquito transmission. Experimental methods commonly used to orally infect mosquitoes in laboratory settings can directly impact virus-vector interactions in at least three ways: (*i*) passage in cell culture can select for virus mutations that are not maintained in nature [6], changing the ability of a virus to infect or replicate in a mosquito [7]; (*ii*) defibrinated blood used in artificial blood meals can alter the distribution of virus in the mosquito midgut and result in a systematic underestimation of infection rates in exposed mosquitoes [8]; and (*iii*) mosquitoes used in experimental infections, often derived from colonies that are many generations removed from field populations, are inbred and may not represent wild mosquito phenotypes and their susceptibility to viruses [9]. Results from these studies indicate varying thresholds of infection for DENV passaged in cell culture in artificially infected, laboratory-colonized mosquitoes, and how these thresholds relate to natural human-to-mosquito transmission are only beginning to be understood.

There have been a few notable exceptions to these traditional methods. In their classic dengue studies, Siler et al. [10, 11] and Simmons et al. [12] demonstrated, through human-to-mosquito-to-human DENV transmission experiments, an intrinsic (human host) incubation period of the virus that generally ranged from 4.5–7 days. Viremia was first detectable 6–18 hours before onset of fever and typically lasted 4–5 days, but could last as long as 12 days. Collectively, people were infectious to mosquitoes from 2 days before to 2 days after the onset of symptoms. *Ae*. *aegypti* that fed on viremic people were able to transmit virus by bite to a second person after ≥11 days of extrinsic (mosquito host) incubation [13, 14]. Later, in 1978, Gubler et al. [15] examined transmission of DENV from humans to mosquitoes by allowing laboratory-reared mosquitoes to feed on the arms of dengue patients in Tonga. With only one participant transmitting to four of ten mosquitoes, it is difficult to draw concrete conclusions from that study. Each of these early experiments was limited to people with clinically apparent DENV infections; i.e., people that sought treatment at a clinic or hospital.

Three recent studies expanded on these findings, examining the infectiousness of humans naturally infected with DENV. In 2013, Nguyen et al. [16] determined rates of infection and dissemination of the four DENV serotypes in *Ae*. *aegypti* fed on participants in a hospital setting in Vietnam. They confirmed the importance of viremia levels (measured in cDNA copies of DENV RNA) to human-to-mosquito transmission and found that an increase in both the IgM titer and day of illness were associated with reduced potential for transmission independently of viremia. In 2016, Tan et al. [17] found that, for 25 participants naturally infected primarily with DENV-1, more mosquitoes were infected (midgut and salivary glands) after imbibing blood from people with higher viremias (measured in viral RNA copies) and infection rates of mosquitoes exposed to ethylenediaminetetraacetic acid (EDTA, an anticoagulant)-supplemented blood delivered through an artificial feeding system was similar to that of mosquitoes fed directly on a viremic person. In both studies, all participants were symptomatic (clinic-captured) cases of dengue.

A third study was carried out in Cambodia, with mosquitoes fed directly on DENV viremic people and neighbors of index cases [18]. DENV-infected participants with no detectable symptoms (i.e., asymptomatic) and those captured before the display of symptoms (i.e., pre-symptomatic) were capable of infecting mosquitoes through direct and indirect exposure and were more infectious than symptomatic participants at the same level of viremia. Similar to the two other recent studies, viremias in this study were measured in cDNA copies of DENV RNA. Extending the methods applied in these studies to explore a broader range of clinical and virological parameters—specifically to include participants with mild or no apparent disease, as in Duong et al., and to quantify viremias using an infectious virus assay—would add important new insights to the study of dengue epidemiology.

The objectives of this study were to (1) compare measures of DENV infection and dissemination in mosquitoes fed directly on humans to those fed on blood collected from the same study participants (blood was untreated and/or EDTA-treated prior to mosquito exposure); (2) correlate these results to measures of infectious DENV particles in blood; (3) obtain preliminary data on the ability of DENV from people with mild or inapparent disease to infect and disseminate within mosquitoes; and (4) optimize enrollment and feeding methods through incremental implementation of a study protocol. A companion report [5] describes human acceptability of direct mosquito feeding based on focus group interviews.

## Materials and methods

### Ethics considerations and participant consent

The study protocol was approved by the US Naval Medical Research Unit No. 6 (NAMRU-6) Institutional Review Board (IRB) (protocol #NAMRU6.2011.0002), which includes Peruvian representation and operates in compliance with all US and Peruvian regulations governing the protection of human subjects. IRB relying agreements were established between NAMRU-6, the University of California, Davis, and the Pasteur Institute. The protocol was reviewed and approved by the Loreto Regional Health Department, which oversees health research in Iquitos. Below, we describe the evolution of our study protocol over time and in response to our observations and recommendations of the NAMRU-6 IRB and Loreto Regional Health Department.

### Participant population

Participants were enrolled from ongoing clinic-based passive and community-based active febrile surveillance studies in the Amazonian city of Iquitos, Peru, an urban center of approximately 400,000 inhabitants where DENV is endemic. Detailed descriptions of the city, its *Aedes aegypti* population, and local DENV transmission are published elsewhere [19–27]. Participants were enrolled between September 2012 and December 2014, when Asian-American DENV-2 was the predominant genotype/serotype in circulation [28]. Below is a summary of each of the parent studies from which participants were recruited.

#### Clinic-based febrile surveillance study

Iquitos residents aged 5 years and older who presented at one of two Ministry of Health hospitals or seven health centers with acute, undifferentiated fever (≥ 38°C or reported use of antipyretics) for 5 days or less were included in this surveillance study [27, 29]. A study nurse introduced the mosquito feeding study at the time of enrollment in the surveillance study and asked if the enrollee was willing to participate in this additional study if they were found to be positive for DENV infection.

#### Community-based cohort studies

Approximately 1,600 households representing approximately 10,000 Iquitos residents were visited 3 times per week for fever surveillance as part of an NIH-sponsored cohort study that began in 2007 [26, 31–34] and a Bill & Melinda Gates Foundation lethal ovitrap trial that began in July 2011. If fever was reported, the affected person was invited to provide acute and convalescent blood samples for dengue diagnosis. As with the clinic-based study, individuals were asked about their willingness to participate in the mosquito feeding study if they tested positive for DENV infection.

#### Contact cluster study

As part of an NIH-sponsored study that began in 2008, our team has carried out many contact cluster investigations [26, 35]. Participants diagnosed with DENV infection were interviewed to determine locations they had visited in the previous 15 days. Residential sites identified were visited, and all residents 5 years and older were invited to provide blood samples for DENV screening by real-time RT-PCR, facilitating the identification of asymptomatic or pre-symptomatic DENV-infected individuals [26]. As above, participants testing positive for DENV infection were asked about their willingness to participate in mosquito feeding experiments.

### Participant enrollment

DENV-positive participants were visited by a study physician, a physical examination was performed, and an invitation to participate in this study (with a video presentation of procedures, see supplementary S1 Video) was offered [5]. As soon as possible, participants were transported to the NAMRU-6 Iquitos laboratory facility, where informed consent was completed, and an additional blood sample was obtained immediately prior to mosquito feeding to determine the DENV titer in blood at the time of direct and indirect feeding procedures. Written informed consent was provided by all adult participants and parent/guardian consents were obtained for all participants under the age of 18. Children aged 8–17 years provided written assent.

**Fig 1:**
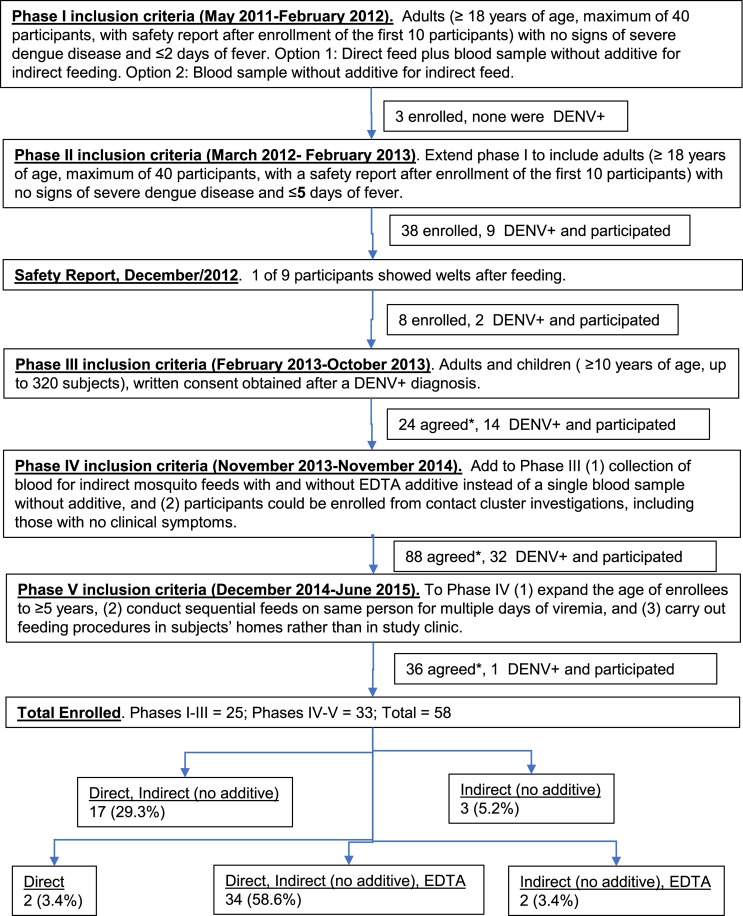
Summary of inclusion criteria for study from initial IRB approval in May 2011 through the end of study in 2015. *During Phase III, written consent was obtained after a DENV+ diagnosis was confirmed, in contrast to Phase I–II, when written consent was obtained prior to diagnosis. In Phase III–IV, participants were asked if they would be willing to feed mosquitoes if they were diagnosed as DENV+. After March 2013, written consent was only required if a participant was DENV+. Here, we note the total number of individuals who agreed to participate in mosquito feeding if they tested DENV+.

In order to develop and assess mosquito feeding methods, the study was conducted in five phases, based on consultation and review by the NAMRU-6 IRB and Loreto Regional Health Department (see Fig 1, flow chart). Through March 2013, participants provided consent before they were determined DENV infected and then were discontinued from the study if they tested negative for DENV. After March 2013, the study was described to participants to gauge interest, but only DENV-positive individuals were asked for consent to participate. Before November 2013 participants were offered direct and indirect-no additive feeding option; after this date, all participants were offered direct, indirect-no additive, and indirect-EDTA. All participants enrolled in this study were monitored by a physician for the five days following enrollment into the parent study or until symptoms resolved. Two DENV-positive participants reported no symptoms at enrollment or in the follow-up days and were considered asymptomatic. Although no participants were hospitalized at the time of enrollment, the full range of illness (from asymptomatic participants to those hospitalized with warning signs) was observed during the follow-up period.

### Clinical monitoring and disease severity scoring

All participants who agreed to the mosquito feeding protocol were evaluated clinically by a nurse technician and project physician. A total of 37 signs and symptoms were recorded for these participants. On the day of enrollment, a participant was asked to recount his/her symptoms on each day since illness began. All symptoms were recorded daily by the project physician, starting with the day of presentation and ending when either all symptoms had disappeared or on day 5 of follow-up if no symptoms were reported. Two scores were calculated: symptom-days and symptom-count. Symptom-days (the number of days participants presented with that symptom during the course of their illness) were summed across all reported symptoms. The symptom-count was a count (+/−) of each disease manifestation (clinical sign or symptom) over the course of illness. For example, for a participant that had 3 days of fever, 4 days of headache, and 1 day of joint pain, their total symptom-days would be 8 and their symptom-count would be 3. We developed a severity score from 0 to 4 using a combination of symptom-days, symptom-count, and hospitalization status, with the following symptoms: bruising, abdominal pain, ecchymosis, hematemesis, blood in stools, bleeding gums, hematuria, hepatomegaly, and splenomegaly. For example, if the participant in the example above had 2 days of abdominal pain and 1 day of bleeding gums, the severe symptom-day score of 3 (severe symptom-count = 2) and overall symptom-day and symptom-count scores would increase to 11 and 5, respectively.

A severity score of 0 was assigned to the two participants who reported no symptoms on the day of clinical evaluation and had a symptom-day score < 5. After scoring, a group associated with very mild illness was found to be distinct from the rest of the participants with more severe illness. This group had a symptom-day score < 40, symptom-count score < 13, and severe symptom count = 0. The remaining participants had severe symptom-counts > 0, symptom-day scores ranging 40–115, and symptom-count scores > 13 (with severity scores of 2 or 3). The highest severity score of 4 was assigned to participants who were hospitalized after participation in mosquito feeds. Most participants continued to be followed while they were hospitalized, and, with the exception of one participant who was hospitalized for hypotension, hospital stays were 2–5 days in duration. All hospitalized participants received close monitoring and fluid treatment. For the development of statistical models (see below), we compared infected mosquitoes that fed on individuals with mild disease (severity score = 1) vs. individuals with more severe illness (i.e., those with clear dengue symptoms, severity scores of 2–4).

### Screening of blood samples for DENV

Whole blood, obtained in Vacutainer tubes containing EDTA, was screened for DENV by two methods. In order to begin study procedures as soon as possible, acute-phase whole blood sample from febrile participants were tested using an NS1 antigen rapid test cassette (NS1 SD Bioline Dengue Duo cassette, SD Diagnostics) according to the manufacturer’s protocol, when the test was available (53 or 58 participants). If a sample tested positive for NS1 antigen, the participant could proceed to mosquito feeding procedures. For a definitive diagnosis of DENV infection, however, serum was separated from whole blood, and all sera were tested by a conventional reverse-transcription hemi-nested polymerase chain reaction (conventional RT-PCR) [36] prior to July 2012, and by real-time reverse-transcription polymerase chain reaction (real-time RT-PCR) [37] after July 2012. When the rapid test was unavailable (5 of 58 participants), the sample proceeded directly to RT-PCR testing as described above.

### Mosquito rearing and testing of parental stock

An F_2_ generation of *Ae*. *aegypti* was used for experimental feeding procedures. This generation was obtained in three stages. Wild-type female *Ae*. *aegypti* adults, obtained through natural oviposition in buckets left in several locations throughout Iquitos and reared to adulthood in an insectary, were fed non-infectious blood meals (chicken blood) through an artificial membrane. Chicken blood was obtained from a local butcher, where blood was available for sale. Resulting F_1_ eggs were hatched in batches of 800 mosquitoes. Adult females were tested for DENV by conventional RT-PCR. No chikungunya or Zika virus transmission was reported during the study. Larvae were reared to adulthood and provided blood meals through a membrane to obtain F_2_ eggs that were hatched and reared in batches of 2,000 for 4–11 days post-eclosion each week for experimental feeding.

### Mosquito feeding and testing of engorged mosquitoes

After blood was drawn, two one-pint containers containing up to 25 lab-reared mosquitoes each were placed on the forearms or calves of participants (based on their choice) for approximately 10 minutes. Blood drawn from the participant was immediately transported to a secure infection room in our insectary, where blood collected in either untreated or EDTA-treated Vacutainer tubes was introduced into glass feeders that were maintained at 38°C with a circulating water bath for exposure to mosquitoes. All mosquitoes used in feeding experiments were 3–5 days old. The remaining sample was transported on ice to the virology laboratory for serum separation and storage at −80°C for subsequent testing. Mosquitoes were maintained under controlled environmental conditions: relative humidity of 70–80% and temperature of approximately 27–28°C. Unfed mosquitoes were maintained in a virus-free room, and blood-fed and potentially DENV-infected mosquitoes were secured in containers in a separate room of the insectary for an incubation period of 13–15 days. Mosquitoes were harvested at the end of this period, and bodies and heads were separated for testing by conventional RT-PCR [34].

### Fluorescent focus assay (FFA)

The dose of infectious DENV in human blood was estimated by FFA for serum in 8-well chambered slides. The assay was later modified to a 96-well sample format as described previously [38]. Briefly, C6/36 cells were seeded in a 96-well plate and grown overnight. The culture medium was removed, and 40 μL of sample were added to each well. After 1 hour, cells were overlaid with a 1:1 mix of cell culture medium (Eagle’s-Minimum essential medium, 0.1% penicillin [10,000 U/mL] / streptomycin [10,000 μg/mL], 1× non-essential amino acids, and 10% fetal bovine serum) and carboxyl methylcellulose, and incubated for 5 days at 28°C. Cells were fixed with 3.7% formaldehyde at room temperature (20–25°C) for 20 min and washed three times with phosphate-buffered saline (PBS). After a first incubation of 30 min at room temperature with 0.3% Triton X-100 in PBS, cells were washed three times in PBS and incubated for 1 hour at 37°C with monoclonal anti-DENV complex D3-2H2-9-21 antibody (produced in-house, NAMRU-6) at 1:50 dilution in 1× PBS and 1% bovine serum albumin (BSA). After another three washes in PBS, cells were incubated at 37°C for 30 min with secondary antibody conjugated with FITC (goat anti-mouse IgG [whole molecule]-FITC antibody, Sigma) 1:100 dilution in PBS and 1% BSA. After three final washes in PBS, infectious foci were counted under a fluorescent microscope and converted to focus-forming units/mL (FFU/mL).

### Statistical analysis

Statistical analyses were carried out separately for feeding (engorgement), survival, infection, and dissemination rates. Analyses of feeding and 14-d survival rates included all 58 study participants, whereas analyses of DENV infection and dissemination included all study participants who had detectable viremia on the day of the mosquito feeding experiment and/or who infected at least one mosquito. Nine of the 58 participants enrolled in the study were excluded from the statistical analyses because they had undetectable viremias or samples were not available from the day of mosquito feeding, with no mosquito that became infected after feeding on that participant’s blood. Statistical analyses of DENV infection and dissemination included 2,120 individual mosquitoes that were each exposed to the blood of one of 49 study participants. The proportion of mosquitoes that had become infected (i.e., body-positive females) and the proportion of infected mosquitoes that had developed a disseminated infection (i.e., head-positive females) were analyzed with a generalized linear mixed model (GLMM) that included the random effect of the participant and marginal fixed effects of gender, age, NS1 test result, day post-onset of symptoms, severity of illness, experimental treatment (direct, indirect-no additive, indirect-EDTA), age, and viremia level as covariates. The GLMM was based on a logit link function and binomial error distribution. Detectable plasma viremia levels were log_10_-transformed prior to analysis. Full models were reduced to minimal adequate models by backward elimination of non-significant terms in a stepwise fashion. Dose-response curves were derived from the logistic regression coefficients. All analyses were performed in the statistical environment R v3.2 [39].

## Results

### Participant characteristics

A total of 197 people consented to directly feed mosquitoes if they subsequently tested positive for DENV, representing about 70% of all febrile individuals invited to participate between May 2011 and June 2015. Of the 58 participants that tested DENV positive and consented to participate in the study (Tables 1 and S1), 53 completed direct mosquito feeding (93.4%) and also provided a blood sample (51 without additive and 34 with EDTA). All infections were with the Asian/American DENV-2 genotype. Only five (8.6%) individuals chose not to directly feed mosquitoes, but blood with and without additive was collected from two of those participants. Sixty-four percent of the participants were male. Ages ranged from 10 to 73 years, with 19 participants under the age of 18 years (32.7%). Most of the feeds were carried out on day 2 (18 of 58, 31.0%) or day 3 of symptoms (22 of 58, 37.9%). Eleven (19.0%) participants were sampled on day 4 and 4 (6.9%) on day 5. Only one participant (1.7%) fed mosquitoes on day 1 of symptoms, and two individuals (3.4%) were asymptomatic at the time they fed mosquitoes.

**Table 1:** Summary of results from mosquito feeding experiments with 58 subjects, including mosquito feeding rates (number engorged of total exposed) and 14-day survival rates of engorged mosquitoes. N represents the number of subjects with data available.

	Engorged (of all mosquitoes exposed to blood)			Survival (of mosquitoes fed to repletion)		
Method	N	Mean (range)	SD	N	Mean (range)	SD
Direct	53	75% (32–100%)	18%	53	66% (19–100%)	22%
Indirect						
Without additive	56	44% (6–96%)	21%	56	71% (6–96%)	23%
With EDTA	36	39% (0–92%)	22%	34	64% (0–100%)	24%

Clinical presentation ranged from asymptomatic to reporting warning signs that led to hospitalization. Thirteen participants received a severity score of 1; these individuals were ambulatory and had a significantly shorter disease duration than the remaining participants. Of other participants, 17, 14, and 12 received severity scores of 2, 3, and 4 (hospitalized), respectively.

### Viremias

Detectable viremias ranged from 1.3 × 10^2^ to 2.9 × 10^6^ FFU/mL. Viremias were not detectable by FFA in 22 participants; yet, of these, 14 participants infected mosquitoes through at least one method (Table 2). Of 18 participants who directly fed mosquitoes but did not have detectable FFA titers, eight (44%) infected mosquitoes, with feedings on four (50%) of these participants resulting in disseminated mosquito infections. Of blood samples without additive, 20 subjects who did not have detectable FFA titers, six (30%) infected mosquitoes. Indirect feeding on the blood of four (67%) of these resulted in virus dissemination to mosquito heads. Of blood samples with EDTA from 14 subjects who did not have detectable FFA titers, seven (50%) infected mosquitoes, and feeding on three of these led to disseminated infections (43%).

**Table 2:** Summary of participants whose blood successfully infected mosquitoes and contributed to disseminated DENV infections, as determined by direct and indirect feeding on blood with detectable and undetectable serum viremias.

Method	Serum titer	No. participants that contributed to	
		Infection of mosquito bodies	Disseminated infections in mosquitoes
Direct	Detectable	28/32^a^ (88%)^b^	21/28 (75%)
	Undetectable	8/18 (44%)	4/4 (50%)
	Not tested	1/3 (33%)	1/1 (100%)
Indirect			
Without additive	Detectable	28/33 (85%)	18/27 (64%)*
	Undetectable	6/20 (30%)	4/6 (67%)
	Not tested	2/3 (67%)	1/1 (50%)
With EDTA	Detectable	14/20 (70%)	9/14 (64%)
	Undetectable	7/14 (50%)	3/7 (43%)

*1 head was not tested

^a^Ratios presented are the number of participants that contributed virus to infected mosquitoes over the number participants that completed that feeding method.

^b^Numbers in parentheses indicate the percentage of all mosquito bodies tested that were determined positive for either infection or dissemination.

### Comparison of direct and indirect feeding methods

#### Feeding efficiency and mosquito survival

Mosquito feeding rates were highly variable regardless of method, but, on average, the percentage of mosquitoes that fed to repletion was significantly higher with direct blood feeding than with artificial feeding on blood either with no additive or with EDTA (Table 1, OR = 3.9, 95% CI 3.5‒4.3, p < 0.0001). Engorgement of mosquitoes that were indirectly fed on blood with no additive was more frequent than that of mosquitoes fed on blood with EDTA (OR = 1.3, 95% CI 1.1‒1.5, p < 0.0001). Mosquitoes that fed to repletion on blood from 57 of 58 participants survived the 13‒15-day incubation period. For one participant, no engorged mosquito survived for analysis. Survival rates at harvest (13–15 days) ranged from 63.5 to 71.2% and were significantly higher for mosquitoes that fed indirectly on blood without additive than for those that fed indirectly on blood with EDTA (OR = 1.4, 95% CI = 1.1‒1.7, p = 0015) or that fed directly on participants (OR = 1.2, 95% CI = 1.0‒1.4, p = 0.0015, Table 1).

#### DENV infection in mosquito bodies

**Fig 2:**
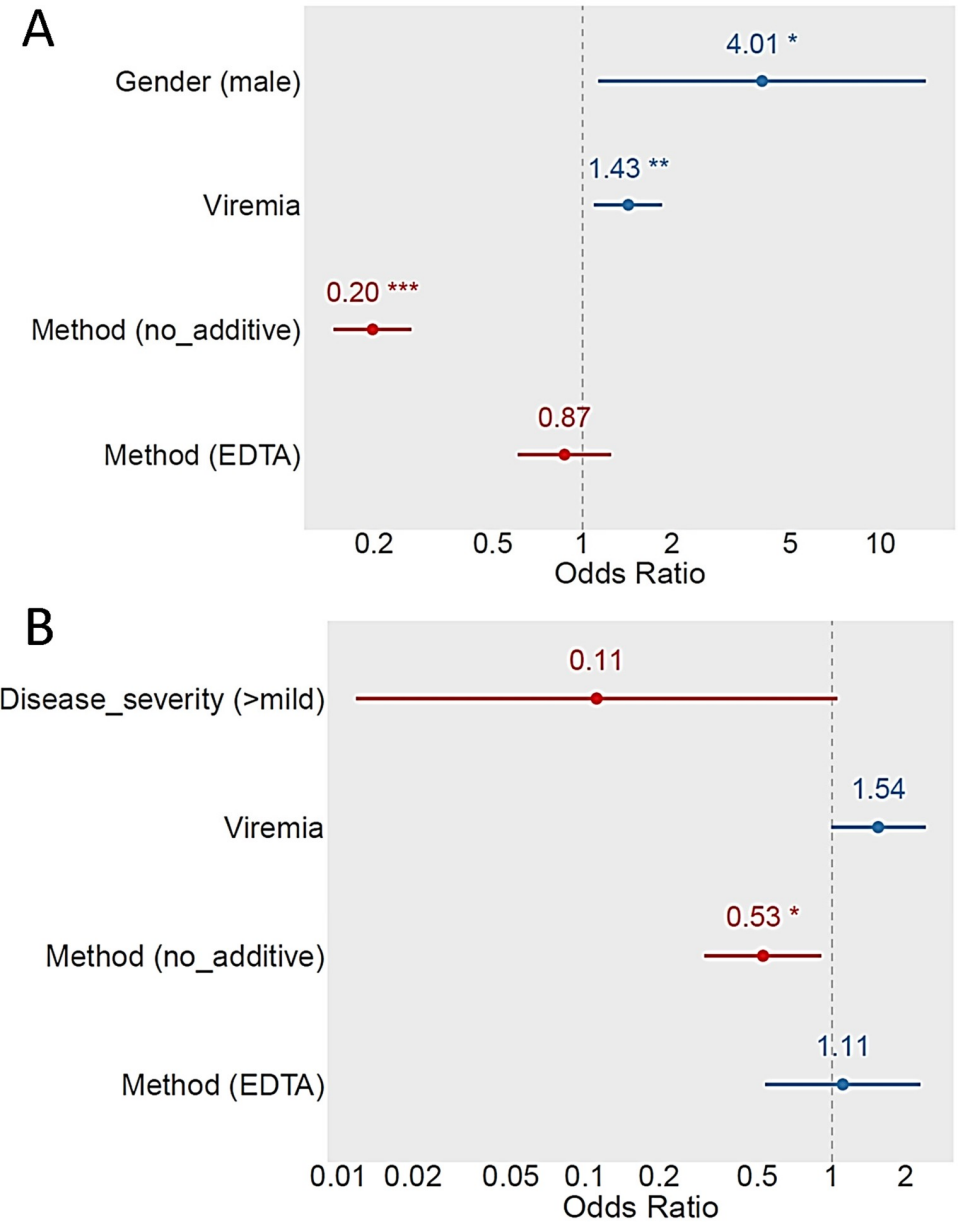
**Significant predictors of mosquito DENV infection (A) and DENV dissemination in infected mosquitoes (B).** Odds ratios (ORs) and their 95% confidence intervals are shown on a log_10_ scale, relative to the reference level, for statistically significant covariates. ORs were calculated from a marginal logistic regression based on a generalized linear mixed model that included the random effect of study participants and fixed effects of covariates. Positive effects are shown in blue, and negative effects are shown in red. Note that in panel B the covariates disease severity and viremia level were marginally insignificant (p = 0.0552 and p = 0.0512, respectively) and therefore kept in the minimal adequate model. Stars represent statistical significance levels: *p < 0.05; **p < 0.01; ***p < 0.001.

**Fig 3:**
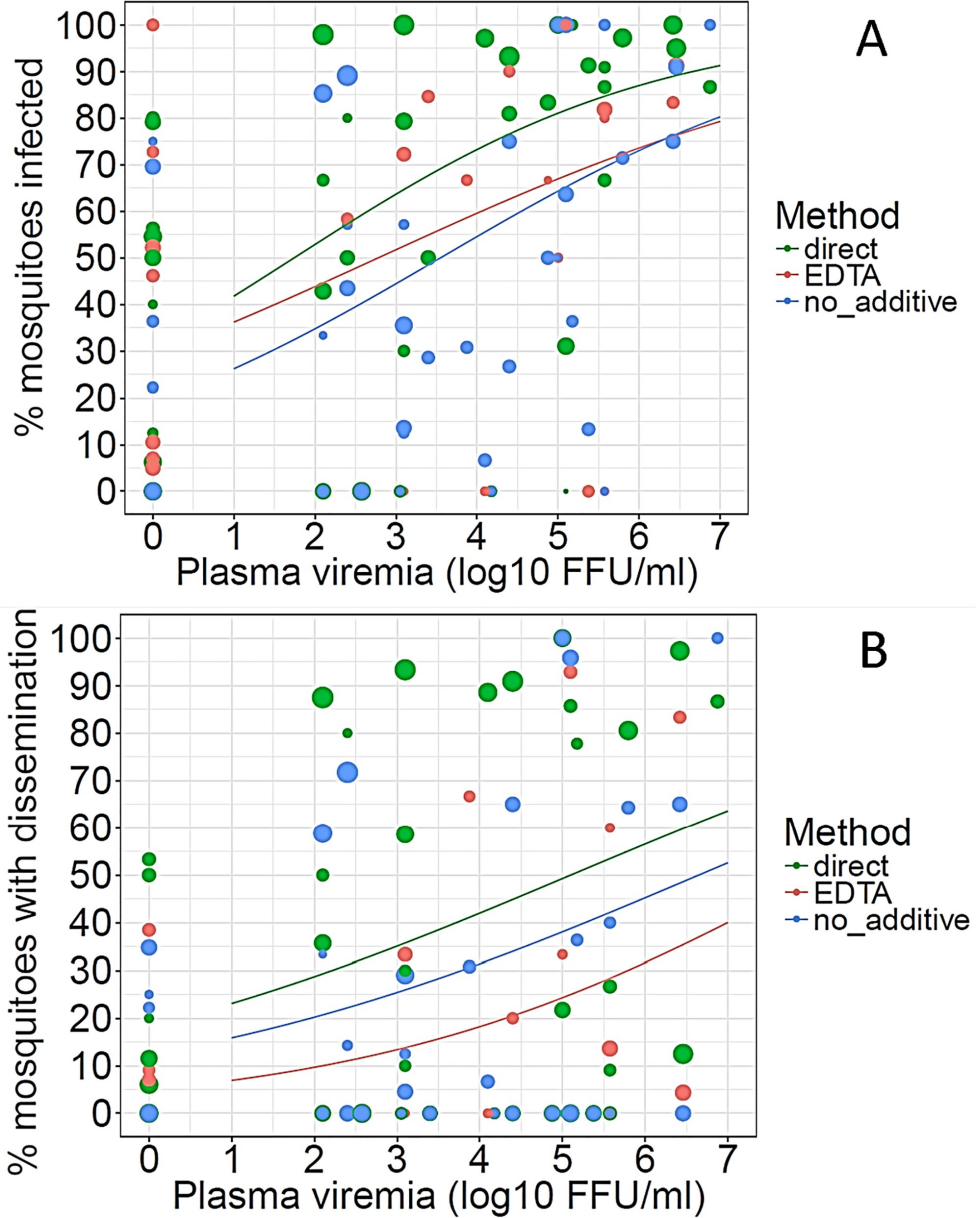
**Dose-response curve of DENV infection in mosquitoes (A) and DENV dissemination in infected mosquitoes (B).** The percentage of infected mosquitoes is shown as a function of viremia expressed in focus-forming units (FFU)/mL of serum for each study participant. Red, green, and blue symbols represent direct feeding, indirect feeding on blood with EDTA, and indirect feeding on blood without additive, respectively. The size of each dot is proportional to the number of mosquitoes tested per participant. Curves are logistic regressions of the data, excluding data for participants with undetectable viremias.

**Fig 4:**
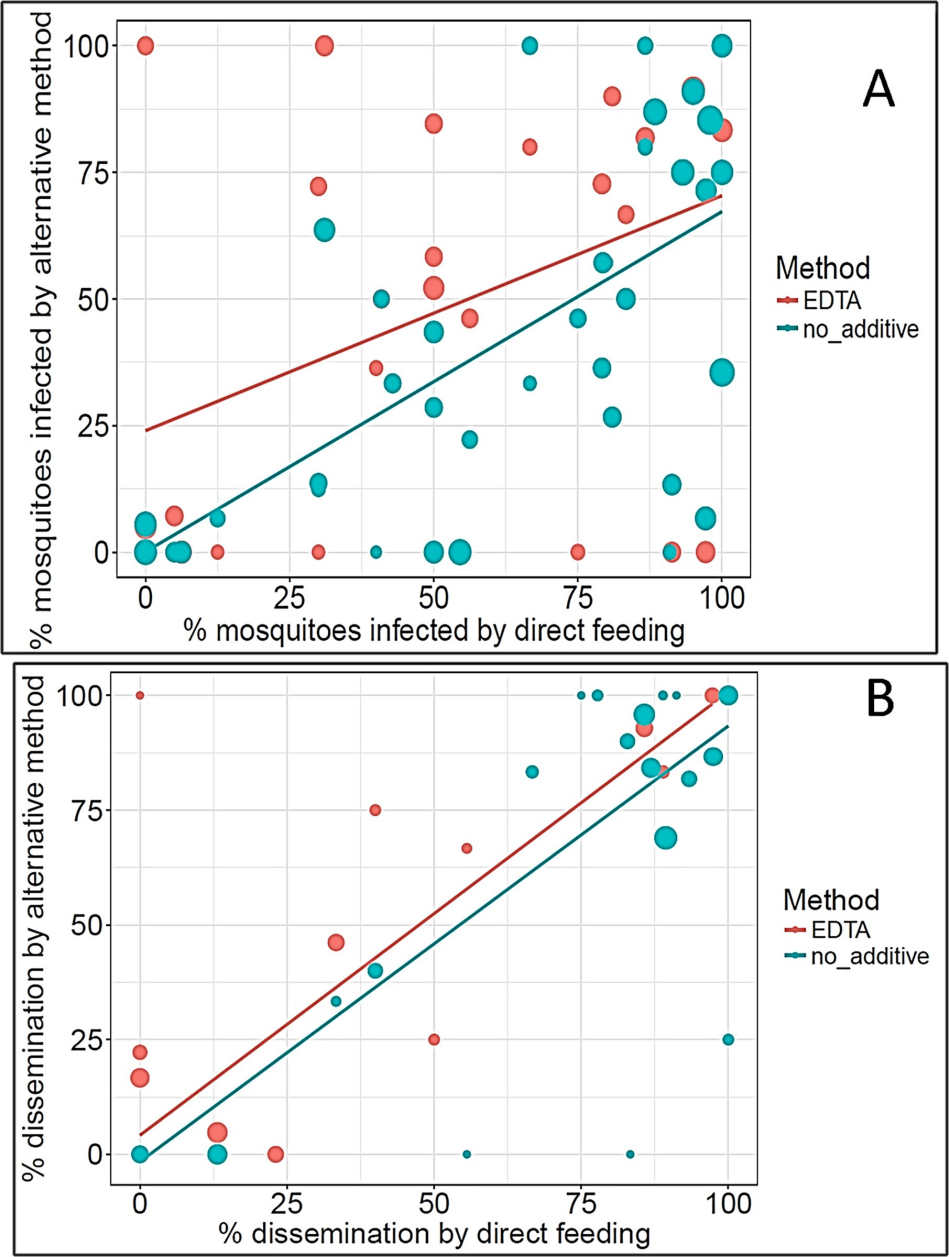
**Correlations in the percentages of mosquitoes showing DENV infection (A) and dissemination (B) following direct and indirect feeding.** Each dot represents the percentage of mosquitoes infected with DENV by direct feeding versus an indirect feeding on the same study participant. Blue dots represent direct feeding versus indirect feeding on blood without additive (infection: R^2^ = 0.48, p < 0.0001; dissemination: R^2^ = 0.83, p < 0.0001). Red dots represent direct feeding versus indirect feeding on blood with EDTA (infection: R^2^ = 0.16, p = 0.0195; dissemination: R^2^ = 0.85, p < 0.0001). The size of each dot is proportional to the total number of mosquitoes tested following feeding by the paired methods. The lines represent weighted linear regressions.

The proportion of surviving-mosquito bodies that tested positive for DENV by conventional RT-PCR varied widely by study participant (S1 Table, S1 Text). Of the participants who had detectable serum viremias at the time of mosquito feeding, 70–88%, depending on the feeding method, infected at least one mosquito (Table 2). Using marginal logistic regression based on a GLMM that included a random participant effect and fixed effects for covariates, the covariates participant age, viremia level, and feeding method (direct, indirect-no additive or indirect-EDTA) were significant predictors of DENV infection in mosquitoes. Indirect feeding on blood without additive was significantly less infectious than either direct feeding or indirect feeding on blood with EDTA (OR = 0.20, 95% CI 0.15–0.27, p < 0.0001, Fig 2A). Although there was no significant difference (OR = 0.87, 95% CI = 0.60–1.25, p = 0.45, Fig 2A) in overall infectiousness between direct blood feeding and indirect feeding on blood with EDTA, the two methods were not equivalent based on a weighted correlation between infection rates (Fig 3). Infection rates following direct and indirect-EDTA blood feeding were poorly correlated (*R*^*2*^ = 0.16, p = 0.0195) due to significant deviations from the regression line (Fig 4). Conversely, despite the overall lower infectiousness of indirect-no additive feeding relative to direct feeding from the same person (Fig 2), the two methods were more strongly correlated (*R*^*2*^ = 0.48, p < 0.0001) because deviations from the regression line were smaller (Fig 4). Infectiousness of blood to mosquitoes decreased slightly with age of the participant, and male participants were marginally more infectious to mosquitoes than females (OR = 4.01, 95% CI = 1.14–14.2, p = 0.0309).

#### DENV dissemination to mosquito heads

Dissemination of DENV to mosquito heads also ranged widely by participant (S1 Table). Disseminated infections were observed, however, in mosquitoes fed on 64–75% of the subjects who successfully infected mosquitoes. Although differences by method among dissemination rates following feeding were less apparent than those of infection rates, similar trends were observed between the two. Indirect feeding on blood without additive resulted in significantly lower dissemination rates than direct or indirect-EDTA feeding (OR = 0.52, 95% CI = 0.31‒0.90, p = 0.02, Fig 2B). As with infection rates, there was no significant difference (OR = 1.09, 95% CI = 0.54–2.28, p = 0.78, Fig 2B) in dissemination rates between direct blood feeding and indirect feeding on blood with EDTA. In mosquitoes with a detectable infection, however, the rates of virus dissemination were highly correlated (EDTA vs. direct: *R*^*2*^ = 0.85, p < 0.0001; no-additive vs. direct: *R*^*2*^ *=* 0.83, p < 0.0001, Fig 4). In addition to the effect of feeding method, disease status and viremia level had a marginally insignificant effect on dissemination rates in the final statistical model. Dissemination rates tended to increase with viremia level and to be lower in mosquitoes fed on people with more clearly apparent symptoms (Fig 2).

## Discussion

Our study was designed to determine whether mosquito feeding experiments could be conducted on a large scale using indirect feeding methods, because we originally considered large-scale direct feeding experiments not feasible. Results from the present study, a study by Nguyen et al. [16], who carried out over 400 direct mosquito feeds on hospitalized patients, and a study by Duong et al. [18], who carried out direct feeds on individuals not showing symptoms at the time of feeding, demonstrate the high acceptability of direct *Ae*. *aegypti* feeding by human volunteers. In our study, of the 53 participants who fed mosquitoes directly, only two reacted significantly, e.g., welts and itching, to the mosquito bites. Tan et al. [17], who compared direct feeding with membrane feeding on blood collected in EDTA tubes from suspected dengue cases, encountered only one of 26 participants who asked to stop direct feeding due to discomfort. Focus group discussions in Iquitos [5] reinforce the notion that direct feeds are acceptable and often preferable to venipuncture by study volunteers in Iquitos. Overall, our study demonstrates the feasibility of using direct and indirect feeding methods for field-based studies on vector competence, extrinsic incubation period, or novel vector control strategies, including vector modification to interfere with virus transmission [40]. Although no single study can demonstrate the feasibility in all contexts, results of this study add to an understanding that, at least in certain contexts, direct feeding is feasible and perhaps preferable to laboratory-based feeding on collected blood samples.

We concluded that direct feeding methods should be used when logistically possible and culturally acceptable, because our results show that neither of the two indirect feeding methods perfectly captured participants’ infectiousness, as determined by direct mosquito feeding. On one hand, infection rates in mosquitoes fed indirectly on blood with EDTA were not significantly different overall from infection rates in mosquitoes that fed directly on the blood of the same participants, but the two methods were poorly correlated due to substantial inconsistencies at the participant level. On the other hand, rates of infection in mosquitoes fed indirectly on blood without additive correlated more strongly with infection rates in mosquitoes fed directly on the same participants, but infectiousness was significantly lower overall for the indirect method without additive. These findings contrast with those of Tan et al. [17], who observed a strong correlation (R^2^ = 0.89) between midgut infection rates in directly fed and EDTA blood-fed mosquitoes. We observed a significantly weaker correlation between rates with these two methods (R^2^ = 0.16). This may be in part due to our use of F_2_ mosquitoes vs. a laboratory strain or on the use of mosquitoes collected in Singapore vs. Peru. In addition, we measured serum viremia levels using an infectious assay (FFA) in contrast to the real-time RT-PCR assay used by Tan et al. [17]. Once infected, mosquitoes fed using any one of the three methods showed similar rates of virus dissemination. Furthermore, dissemination rates were highly correlated between the feeding methods, consistent with the findings of Tan et al. (14). After feeding method, viremia level was the most important predictor of infection and dissemination.

Our results indicate that infection is possible at DENV titers that were undetectable by our FFA. The theoretical limit of detection of our FFA is 2.5 x 10^1^ FFU/mL and this was consistent with the lowest detected titer of 1.3 × 10^2^ FFU/mL. Twenty-two of the participants in our study did not have detectable titers of DENV at the time they fed mosquitoes or provided blood samples for feeding. This indicates that the FFA titer in venous blood might be an underestimation of capillary/veinual blood viremia. Based on the assumption that a mosquito imbibes about 2 μl of capillary blood, the minimum viremia is 5 x 10^2^ FFU/mL for a 2-μl blood meal to contain at least 1 FFU. According to this calculation, the minimal mosquito infectious dose (5 x 10^2^ FFU/mL) should always be detectable by FFA (limit of detection: 2.5 x 10^1^ FFU/mL). Our observations support the hypothesis that freshly engorged mosquitoes (containing capillary blood) might be more relevant to estimate viremia than venous blood in infectiousness studies.

Based on our results, indirect feeding methods did not fully replicate direct feedings to provide information on the potential for DENV transmission from humans to mosquitoes. In our experience, and those of prior researchers, direct mosquito feeding appears to be superior in terms of logistical ease, biosecurity, and efficiency. Which of the two indirect methods is preferable is not certain, but indirect feeding with EDTA is the more logistically feasible approach and results in similar (although variable) infection rates compared to that from direct feeding. Based on information from focus groups [5], transport of mosquitoes in secure netted containers in fitted boxes to participant homes can enhance participation in the feeding protocol, compared to mosquito feeding in the laboratory. Direct feeding experiments are now conducted in participants homes in an ongoing protocol being carried out by our research team in Iquitos since 2015. Although blood samples are routinely handled in the laboratory, introducing blood into the mosquito feeder and cleaning materials after feeding requires additional biosafety and logistical measures. A higher percentage of mosquitoes consistently fed to repletion during direct exposure to participants than when they fed on blood through an artificial feeder. Direct feeds, therefore, allow for more efficient use of mosquitoes reared for experimental procedures.

Our study illustrates that through incremental project roll-out, researchers, IRBs, and participant communities can work together to examine issues of safety and acceptability [5] when developing new public health research methodologies. Through step-wise review and implementation, we expanded our inclusion criteria of participant age (reducing the minimum age from 18 to 5 years), disease profile (expanding the range of disease severity to include the full spectrum of dengue illness), and number and location of feedings (expanding to multiple sequential feeds on the same person and feeding in participants’ homes, rather than in a clinic). Our approach recognized and addressed participants’ interests, while also establishing a platform for a more natural assessment of human-to-mosquito virus transmission.

A variety of factors determine which people transmit DENV to mosquitoes and when during their infectious period transmission is most likely to occur [18, 41]. These factors influence the dynamics of DENV transmission and, thus, shape dengue epidemiology. In future studies, we aim to capture DENV-infected participants earlier during their infectious period and to feed mosquitoes on participants multiple times during the course of their infection to better define the temporal dynamics of human infectiousness to mosquitoes and to refine understanding of important properties of mosquito-virus interactions, such as the duration of the intrinsic incubation period.

## Supporting information

S1 Video Consent video presented to participants at enrollment in mosquito feeding experiments corresponding to Fig 1 in manuscript. Video in wnv format.
(WMV)

S1 Table Data summary of mosquito blood feeding experiments on 58 human participants. DoI = Day of illness; Viremia (log 10) expressed in FFU/ml, IR = infection rate (PCR+ bodies/total tested); DR = Dissemination rate (PCR+heads/PCR+bodies), SC = Disease Severity category (1 = mild, 4 = hospitalized, 2–3 = intermediate scores (see methods), and EXP = exposed.
(XLSX)

S1 Text Description of final data cleanup for statistical analysis. Explains participants from S1 Table that were excluded from statistical analysis.
(DOCX)
