# Supplementary material for: Feasibility of feeding Aedes aegypti mosquitoes on dengue virus-infected human volunteers for vector competence studies in Iquitos, Peru
Source: PLoS Negl Trop Dis. 2019 Feb 12;13(2):e0007116. doi: 10.1371/journal.pntd.0007116 (PMC6388938; doi:10.1371/journal.pntd.0007116)
Supplement: S1 Text — Explains participants from S1 Table that were excluded from statistical analysis. (DOCX) [file pntd.0007116.s003.docx]

- Started with dataset of N=2,527 mosquitoes and N=58 MOSIDs
- Removed 1 mosquito with missing data for both head and body (OK)
- Removed 8 MOSIDs with TITER=0 and mean(BODY)=0
  - MOS0006, MOS0041, MOS0091, MOS0093, MOS0100, MOS0106, MOS0111, MOS0114
- Removed MOS0029 with missing TITER and mean(BODY)=0 (25 mosquitoes)
- Removed 8 mosquitoes with BODY=0 and HEAD=1
- Ended with dataset of N=2,120 mosquitoes and N=49 MOSIDs

| Analysis Variable : irate |  |  |  |  |
| --- | --- | --- | --- | --- |
| TREATMENT | N Obs | Mean | Std Dev | Std Error |
| D | 44 | 57.6096514 | 36.8178967 | 5.5505068 |
| E | 30 | 45.1507221 | 39.0070183 | 7.1216746 |
| I | 49 | 38.9245731 | 35.1647954 | 5.0235422 |

| Analysis Variable : drate |  |  |  |  |
| --- | --- | --- | --- | --- |
| TREATMENT | N Obs | Mean | Std Dev | Std Error |
| D | 35 | 52.170224 | 40.6354506 | 6.8686448 |
| E | 19 | 33.2979886 | 39.5245572 | 9.0675553 |
| I | 29 | 51.265649 | 41.6946535 | 7.8795489 |

| Analysis Variable : dsrate |  |  |  |  |
| --- | --- | --- | --- | --- |
| TREATMENT | N Obs | Mean | Std Dev | Std Error |
| D | 35 | 41.0853861 | 37.4942231 | 6.3376804 |
| E | 19 | 23.9505498 | 30.8233324 | 7.0713574 |
| I | 29 | 34.4896282 | 33.6927113 | 6.3673239 |
